# Spatio-Temporal Variations of Zooplankton and Correlations with Environmental Parameters around Tiaowei Island, Fujian, China

**DOI:** 10.3390/ijerph191912731

**Published:** 2022-10-05

**Authors:** Zhi Zhang, Zhizhou Shi, Zefeng Yu, Konglin Zhou, Jing Lin, Jiangyue Wu, Jingli Mu

**Affiliations:** 1 Fujian Key Laboratory on Conservation and Sustainable Utilization of Marine Biodiversity, College of Geography and Oceanography, Minjiang University, Fuzhou 350108, China; 2 Joint Research Center of Marine Ecology of Coastal NPP, Ningde Marine Environmental Monitoring Center of State Oceanic Administration, Ningde 352100, China; 3 Key Laboratory of Marine Ecological Monitoring and Restoration Technology, Ministry of Natural Resources, Shanghai 201206, China; 4 Fujian Ningde Nuclear Power Co., Ltd., Ningde 355200, China; 5 National Marine Hazard Mitigation Service, Ministry of Natural Resource of the People’s Republic of China, Beijing 100194, China

**Keywords:** zooplankton, spatio-temporal distribution, copepods, jellyfish, Tiaowei Island

## Abstract

The present study illustrates zooplankton dynamics in relation to environmental factors from the surrounding area of Tiaowei Island based on ten seasonal sampling cruises over three years. A total of 116 species of zooplankton were collected with a predominance of Copepoda (mainly consisting of Centropagidae, Oithonidae, *Acartia*, *Labidocera* and *Paracalanus*), accounting for 31.6 % of the total number of species. The diversity indices indicated a relatively high richness, abundance and evenness of zooplankton ranging from 2.794 to 4.012 on the Shannon–Wiener index for each cruise. More than 20 species of Cnidaria medusae are found as gelatinous organisms, which not only compete with fish but also potentially cause disasters. Significant seasonal variations were detected in both the zooplankton structure and environmental variables. NMDS illustrated a highly overlapping community structure in spring, autumn and winter, while the zooplankton composition in the summer was different from that of the other three seasons with a higher diversity index. Meanwhile, out of thirteen environmental parameters, eight varied significantly among seasons but there were no significant variations among stations. The biota–environmental relationship following a redundancy analysis revealed that water temperature, pH, salinity, dissolved oxygen and suspended particulate composition were the main environmental parameters, seasonally impacting the zooplankton communities. Planktonic larvae (such as nauplius larvae and branchyura zoea) and some zooplankton (including *Corophium sinensis* and *Oithona* *similis*) were significantly vulnerable to the dynamics of suspended particulate composition and water temperature.

## 1. Introduction

Understanding the composition, distribution and dynamics of the biodiversity and biomass shaping community structure is a central theme in ecology [1]. Evaluating the community assembly of organisms and determining how environmental factors interact to shape biological assemblages has practical implications for natural resource management policy and species conservation [2,3]. Sufficient and high-quality water is important for supporting the development of human society and maintaining the integrity of ecosystems [4,5]. Among the aquatic organisms, zooplankton are found in almost all kinds of waterbodies, which plays a vital role in marine ecosystems by redistributing nutrients and regulating biogeochemical cycles [6,7]. Compared with other aquatic organisms, zooplankton species are small in size, numerous in quantities and strong in metabolic activities [8]. Zooplankton play a crucial role in not only the material transport and energy flow through the food web but also in the control of the community structure and population size in different aquatic biomes via upward and downward effects [9,10].

However, the composition of zooplankton species is susceptible to environmental changes, and they can respond quickly to rapid environmental changes, including physicochemical, biological and microbiological parameter changes [11,12]. For instance, zooplankton communities can be used as indicators to evaluate the potential influence of eutrophication [13,14,15], acidification [16,17], global warming [6,18,19] and even ultraviolet radiation [20]. In particular, the abundance and compositions of phytoplankton and Copepoda are used as ecological indicators to investigate the effect and early process of eutrophication in a localized area [14]. Hence, zooplankton was chosen to monitor the impact of environmental change and industrial construction.

Tiaowei Island, located in the north of Fujian Province in southeastern China, is adjacent to the Zhejiang fishing grounds in the north and the Taiwan Strait in the south, which is a subtropical sea area with rich fish resources and other marine organisms [21]. However, relatively low biodiversity of fish and benthos has been reported near Tiaowei Island in recent years [21,22], which might be related to the anthropogenic influence. More dramatically, land reclamation and other construction have linked the island to the mainland in recent years, resulting in changes in the hydrological and ecological statuses. However, as the intermediate link between primary production and nekton [23], the composition and structure of zooplankton are still lacking. In addition, Deng et al., (2020) described the distribution of giant medusas in the adjacent area, suggesting the potential ecological risk of a jellyfish bloom [24]. Hence, it is necessary to conduct a systematic survey of the zooplankton structure in this area, as well as to investigate the biota–environmental correlation, to understand the impacts of development on the surrounding environment. In the present study, we aim to understand the zooplankton composition, their spatio-temporal variation and their relationship with environmental factors. The results will provide a scientific basis for fishery management and offshore industrial development.

## 2. Materials and Methods

### 2.1. Sampling, Identification and Classification

**Figure 1:**
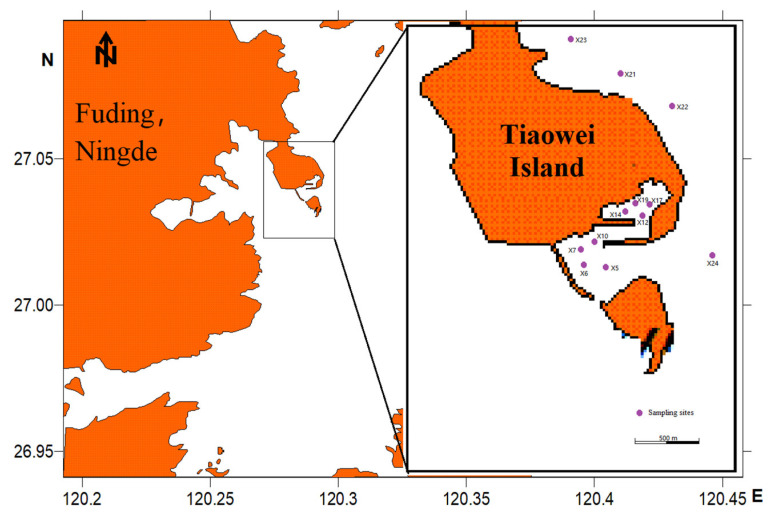
Sketch map of the sampling sites.

In the present study, sixteen sampling stations from the surrounding waters of Tiaowei Island (120.28° E~120.30° E, 27.03°~27.04° N) were chosen to seasonally collect zooplankton samples during ten cruises from January 2018 to December 2020 (Figure 1). Zooplankton were sampled with two types of conical plankton nets: 505 μm mesh size (0.5 m mouth diameter) and 160 μm mesh size (0.316 m mouth diameter), which were towed vertically at ~1 m/s from near the sea bottom to the surface (0~50 cm depth of water column). Samples for species enumeration and identification were preserved in 5% neutralized formalin seawater solution.

Back at the lab, the zooplankton were all counted. A portion of the samples was split into 1/2 to 1/5 subsamples, and all species were enumerated using a Folsom plankton splitter. The organisms were counted and identified at the lowest taxonomic level, such as genera and species, using a stereomicroscope. Some groups (e.g., larval forms), however, were only identified into major taxonomic groups. According to the abundance, a portion of the samples was split into 1/2, 1/4 or 1/8 subsamples using a Folsom plankton splitter to count the dominant species, and all species were classified and enumerated in all of the samples. In general, the dominant taxa, such as Copepoda and Cladocera, were identified to the genus or species levels. The taxonomy of the zooplankton species follows the World Register of Marine Species (WORMS) database (http://www.marinespecies.org (accessed on 25 April 2021)) and was determined using the main taxonomic references available [25].

### 2.2. Measurements of Environmental Variables

Surface water temperature, salinity, pH and dissolved oxygen (DO) were measured in situ with a handheld multiparameter instrument (Pro Plus, YSI, Yellow Springs, OH, USA). Surface water samples were collected from a 0.5-m depth using Niskin bottles. Water samples for nutrient analyses and chlorophyll-a measurement were collected and preserved according to Rizzo et al. (2020) [26]. The measured dissolved inorganic nutrients included nitrate (NO_3_—N), nitrite (NO_2_—N), ammonium (NH_4_—N), phosphate (PO_4_—P) and silicate (SiO_4_—Si). Chlorophyll-a (Chl-a) was analyzed using a simplified method by Sartory and Grobbelaar (1984) [27]. The suspended particulate’s composition was detected by a semi-analytical approach following Sun et al. (2013) [28]. The chemical oxygen demand (COD) was determined by the fast digestion-spectrophotometric method following Alexandra et al. (2011) [29].

### 2.3. Statistical Analyses

Measures of zooplankton community diversity take into account the richness of zooplankton species as well as the degree of disturbance to ecological communities due to environmental parameters. By combining the Shannon–Wiener diversity index (*H*′), the Margalef richness index (*D*) and the Pielou index (*J*′), a comprehensive analysis of community structure can be performed in order to avoid the bias caused by using a single index. *H*′ and *D* indicate the complexity of the community structure, whereas *J* reflects the maturity and stability of the community. All of the above diversity indices were calculated in the Vegan package in R software [30].

Discrepancies and patterns in the zooplankton community among stations and seasons, respectively, were detected and visualized using a non-metric multidimensional scaling (NMDS) ordination based on weighted-average hierarchical cluster analysis (Bray–Curtis similarity index). A one-way analysis of similarity (ANOSIM) was carried out to determine the difference between zooplankton assemblages and voyages, stations, seasons and years, respectively. A similarity of percentage analysis (SIMPER) was conducted to identify species that contributed most to the spatial or temporal dissimilarities of zooplankton assemblages [31]. All of these analyses were also conducted using the Vegan package in R.

The one-way ANOVA of environmental variables through different groups consisting of 4 seasons or 16 stations was conducted to test whether there was a significant variance between stations or seasons. Environmental variables were subjected to a principal component analysis (PCA) to determine the general relationship between environmental variables and figure out the major environmental factors contributing to the variation among seasons or stations. To determine the possible relations between the biological and environmental data, Pearson’s correlation analysis was firstly performed to evaluate the influence of the environmental parameters on the zooplankton abundances. All of the statistical analyses were performed using IBM SPSS (version 20.0). Based on the detrended correspondence analysis (DCA), the maximum gradient length of the axis did not exceed three standard deviations. Thus, a redundancy analysis (RDA) was employed to discover the relationship between zooplankton and environmental factors. All of the species abundance data were log(x + 1) transformed for normalization. During the RDA analysis, a Monte Carlo simulation was employed to test the significance of environmental parameters in explaining the zooplankton abundances under an unrestricted model of 999 permutations.

## 3. Results

### 3.1. Environmental Factors

The variations in physical and chemical characteristics are shown in Table 1. No significant spatial differences (ANOVA, *p* ≥ 0.05) were found in these variables. In contrast, the temporal differences were exhibited in salinity, pH, DO and all dissolved inorganic nutrients (NH_4_-N, NO_3_-N, NO_2_-N, PO_4_-P and SiO_4_-Si). This illustrates that the WT and salinity were persistently declining, while the NO_2_-N, Chl-a and suspended particulate show a trend of increasing after falling in one year (Table 1). Spatially, although no significant difference was detected, the variation in water temperature was unusual with great fluctuations at different stations.

**Figure 2:**
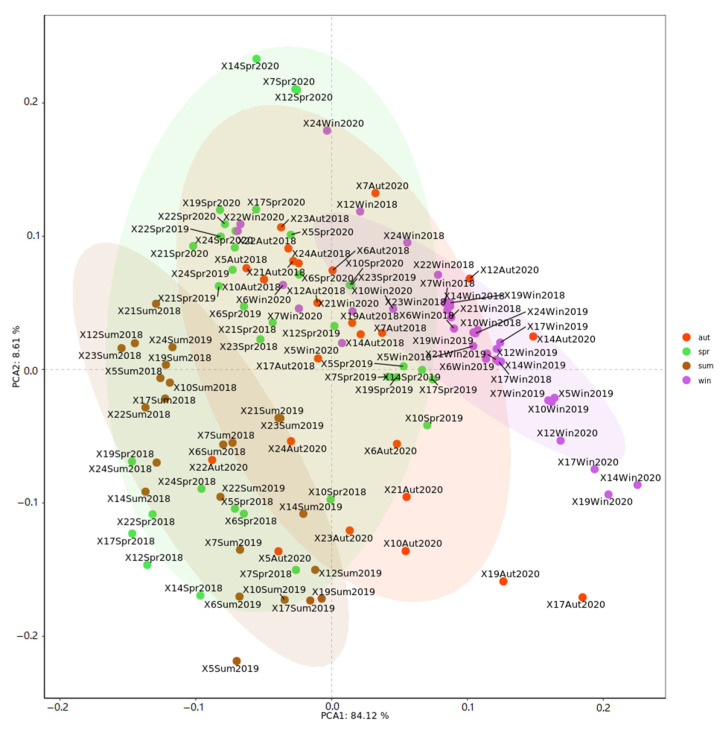
PCA ordination plot of ecological parameters in four seasons.

The PCA identified two principal axes that explained 92.7% of the variation in environmental variants (Figure 2). Axis-1 explained 84.1% of the variation. It was positively correlated with SiO_4_-Si and NH_4_-N and negatively correlated with water temperature and NH_4_-N. Axis-2 explained 8.4% of the variation and was positively associated with Chl-a. From observation of the seasonal trend, the range of environmental variations in spring and autumn is much more extensive than that in summer and winter. Factors including water temperature, salinity, pH and Chl-a are attributed to the differences between summer and other seasons. Alternatively, NO_3_-N and PO_4_-P accounted for the variation in winter.

### 3.2. Species Composition and Diversity Index

#### 3.2.1. Zooplankton Composition

**Figure 3:**
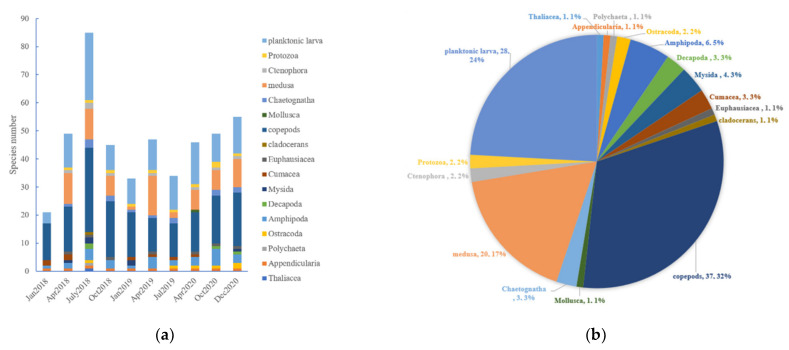
Species composition and classification (**a**) in each voyage (**b**) of the total.

A total of 116 species of zooplankton belonging to 84 genera from 11 phyla, 22 classes, 41 orders and 71 families were identified via microscope from the samples collected over 10 cruises (Figure 3a). The zooplankton consisted of 37 species of Copepoda (accounting for 31.6 % of the total number of species), whereas the second species-rich group was that of Cnidaria medusae with 20 species (17.1%) (Figure 3b). Additionally, abundant planktonic larvae were found in this area (28 types belonging to 18 orders, 11 classes and five phyla), so we treated them as a separate group. Most Copepoda belonged to 21 genera of four orders. Brachyura zoea, *Oithona* *similis* and *Paracalanus* *crassirostris* were identified as the dominant species; in total, these were detected in more than 70% of the samples.

#### 3.2.2. Variations in Abundance and Diversity Index

From observation of the diversity indices, significant differences were observed including species number (*S*), the Shannon–Wiener diversity index (*H*′) and the Margalef richness index (*D*), but no difference in the Pielou’s evenness index (*J*′) was observed among the cruises (Table 2a). *D* ranged from 4.702 (January 2018) to 12.411 (July 2018) with an average of 7.953. The co-variation tendencies were illustrated between *D*, *S* and *H*′, in which *D* had the most obvious change. Among the stations (Table 2b), only *S* and *D* showed a significant difference that X5 had the highest diversity (*H*′ = 4.134), and X24 had a less abundant composition (*S* = 54).

### 3.3. Spatial and Temporal Variations in Zooplankton Communities

**Figure 4:**
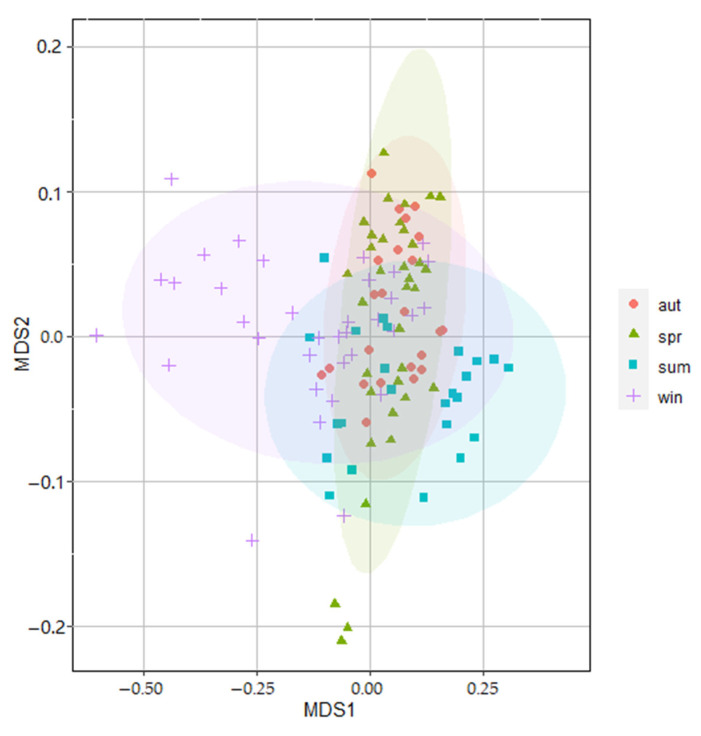
NMDS ordination plot of seasonal changes of zooplankton assemblage structure around Tiaowei Island based on the Bray–Curtis similarity of zooplankton taxa.

Overall, the assemblage structure varied non-significantly in terms of the temporal and spatial scales. The ANOSIM showed that the zooplankton assemblages differed significantly only among seasons (Global R = 0.063, *p* = 0.004 < 0.05). However, there were no differences among different cruises (Global R = 0.063, *p* = 0.05), years (Global R = 0.049, *p* = 0.07) or stations (Global R = 0.067, *p* = 0.14) with a low global-R value and all *p*-values of more than 0.05. Furthermore, the NMDS ordination plot confirmed the seasonal difference in the zooplankton community structure. Except for summer, the distributions of the community structure in the other three seasons were highly overlapped (Figure 4). Finally, according to the SIMPER analysis, zooplankton assemblages were highly dominated by copepods. No significant changes in the zooplankton assemblages in cruises or stations were confirmed by the NMDS of the substantial overlap within the community structures of these groups.

Specifically, at the temporal scale, the structure of assemblages varied more obviously that the seasonal differences described in the above ANOSIM and NMDS results. In addition, the variations of the species richness and compositions among the samples from different cruises were significantly enormous; 80 species were identified in the summer of 2018, whereas 22 in the winter of 2018 (Table 2a). Seasonally, in terms of specific taxa, the number was the highest in summer, while the decrease was not evident in spring and winter. In autumn, the number of taxa was lower than in the other three seasons, especially for the sharply decreasing food-resource organisms, such as copepods and some arthropods. Except autumn, fish eggs and larvae were collected in the other three seasons. Spatially, there was no significant difference in the zooplankton community structure among the stations. Although there were some differences in the numbers of taxa and the occurrence rates of the spatial distributions, there were no significant differences in the compositions of dominant taxa, nor the compositions of the larvae of fishes and zooplankton.

### 3.4. Relationships between Zooplankton Assemblage and Environmental Factors

**Figure 5:**
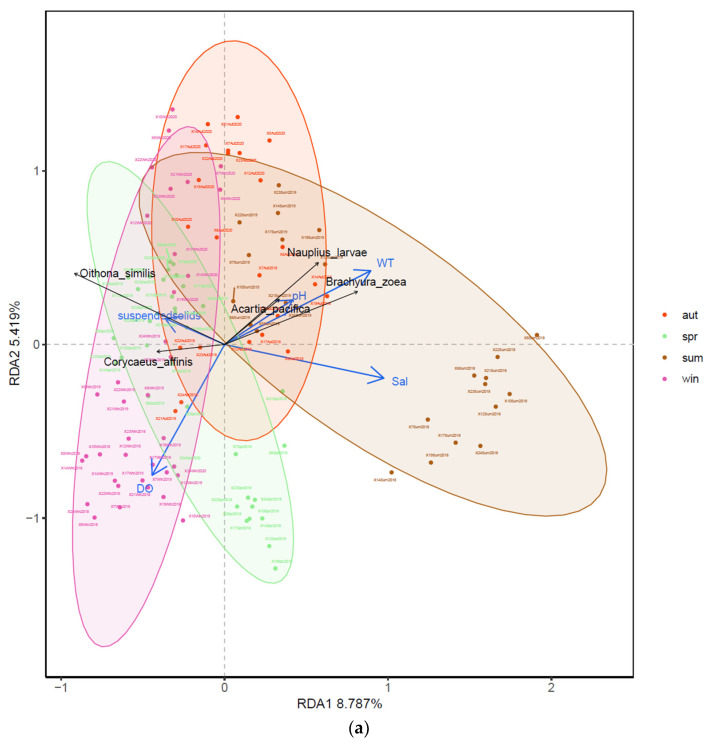

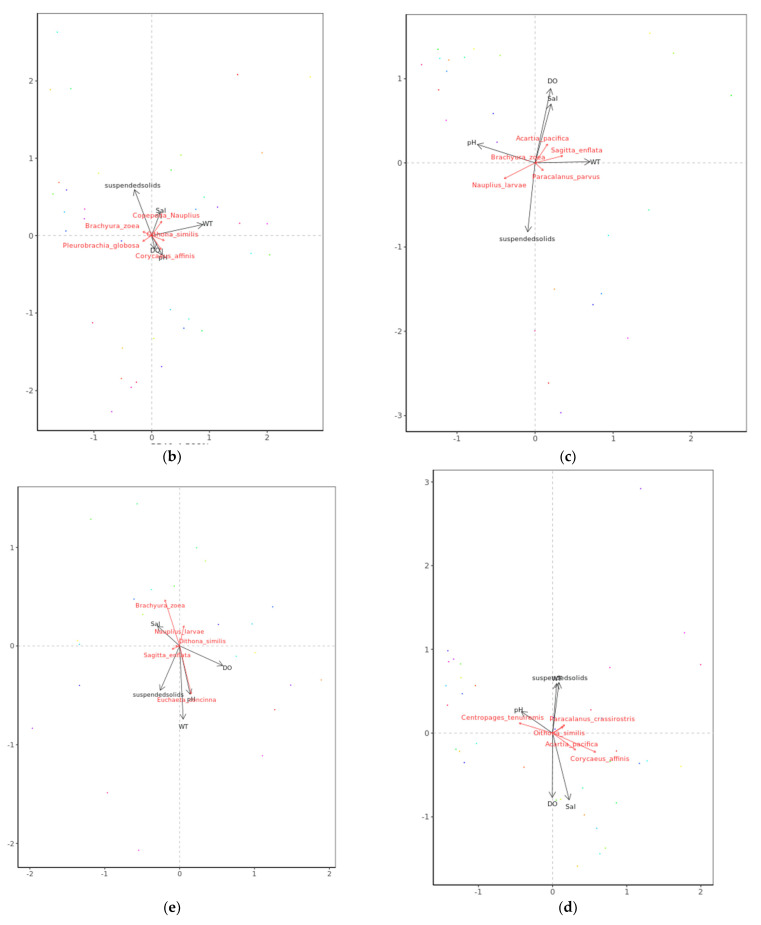
RDA ordination plots showing relationship between zooplankton communities and environmental variables (**a**) in all cruises and (**b**–**e**) during four seasons. The blue lines with arrowhead showed the five main environmental factors, while black (**a**) or red (**b**–**e**) lines illustrated the top five species in zooplankton community structure affected by the environment. (**b**–**e**) represented spring, summer, autumn and winter, respectively.

The RDA showed the relationship between the zooplankton community structure and environmental factors. The Monte Carlo test was significant for the first axis and all canonical axes (*p* < 0.001), suggesting that these environmental variables are important factors in explaining the group compositions. The eigenvalues for RDA axis 1 (0.624) and axis 2 (0.058) together explained 68.2% of the species variance (Figure 5a). Figure 5b–e illustrated the main environmental factors affecting the zooplankton assemblages in different seasons. The factors affecting the seasonal variations in the zooplankton assemblage were water temperature, salinity, pH, DO and suspended particulate composition. Water temperature was the most important factor. Planktonic larvae (such as nauplius and Brachyura zoeae) and some copepods (*C. sinensis* and *O. similis*) were positively correlated with environmental changes in each season. A positive correlation between the planktonic larvae and the water temperature was found in the present study, while the concentration of suspended particulate composition showed a strong positive correlation with some large-size zooplankton, suggesting that abundant organic debris was an important feeding ground for them.

## 4. Discussion

### 4.1. Species Composition

A total of 116 zooplankton taxa were classified during 10 cruises over three years. Abundant zooplankton with high biodiversity existed around Tiaowei Island. The sampling sites belong to nearshore water, but the number of species is similar to other surveys in the Taiwan Strait [32,33]. The long duration and high frequency of the sampling cycle contributed to obtaining abundant diversity data. In the present study, the major group mainly included several Cyclopoida (mainly including *Corycaeus affinis* and *Oithona similis*) and Calanoida (mainly including *Paracalanus crassirostris*, *Acartia pacifica*, *P. parvus*and *Subeucalanus subcrassus*), which was consistent with zooplankton structures from other Chinese coasts [25]. Meanwhile, these copepods are widely distributed in the East China Sea and Taiwan Strait [33,34,35,36,37], suggesting regional distribution characteristics and a local community structure of zooplankton. The dominance of copepods in the zooplankton community was well-documented in various estuaries across the world in both tropical and temperate regions [14,38,39], indicating that their composition can be an important bio-indicator for environmental monitoring. Among copepods, *Oithona* copepods showed the largest abundance in the zooplankton community in this research. They seem to be a key element and can be regarded as the largest genus in the zooplankton community [40,41,42,43]. The *Oithona* species occurs in almost any marine environment, which is possibly due to its euryhaline and eurythermal characteristics [44,45]. Hence, *Oithona* was the main contributor to the copepod abundance in temperate and subtropical seas, which corroborates with the present study [14,45].

### 4.2. Variation in Diversity Indices

Diversity indices can serve as a good indicator of an ecosystem. A high diversity index (*H’*) indicates a healthy ecosystem, while a low value denotes a less healthy or degraded ecosystem, and a fall in the *D* value shows a rise in the level of eutrophication [12,46]. In this study, the average zooplankton diversity indices for all stations or cruises were high, implying that the ecosystem in the surrounding area of Tiaowei Island is relatively healthy. However, some potential threats and changes in the zooplankton composition are also worthy of attention. In addition to the potential risk of disaster, the large number of large-size medusae will inevitably compete with fishes and other nektons [47,48]. The biodiversity of fish is also reported to be lower than surrounding seas, which supports medusae seizing the food resources and territory of fish as the victorious competitors [23]. Meanwhile, the dominance and wide distribution of copepods, with the spawning environment of the surrounding area, suggest the potential of this area as a spawning and feeding ground for protection [49,50].

### 4.3. Spatial-Temporal Dynamics of Community Structure

Spatial and temporal differences in the zooplankton were observed in the taxonomic composition, diversity index and NMDS analysis. The number of taxa in autumn was much smaller than that in the other three seasons (Table 2a). In particular, the abundance of food resources decreased sharply, which may be caused by the consumption of high-level consumers. Most of the predators feed on a large amount of prey before the onset of winter and migration to the wintering grounds, which also requires a lot of energy, so the number of food resources in autumn decreases sharply [51,52]. Moreover, the lack of fish egg collection in the autumn and summer of 2019 suggests a tendency to move the spawning time forward. Spatially, we found that there was no significant difference in the zooplankton composition and diversity index among the different stations (Table 2b). The sampling sites are relatively similar to each other. Nevertheless, no spatial differences in the community structure also indicated that the zooplankton communication at various stations had not been found to be affected by nearby engineering construction.

### 4.4. Relationship between Environmental Parameters and Zooplankton

Organisms are someway sensitive to environmental factors. In particular, zooplankton and phytoplankton, the tiniest creatures in the ocean, are more susceptible to physical and biological factors [13,53]. First of all, for the spatio-temporal changes in environmental factors, the PCA showed that only seasonal differences were significant. Special attention should be paid to thethe negative correlation between NH_4_-N and water temperature, while a positive correlation between NH_4_-N and SiO_4_-Si. Since SiO_4_-Si can only be imported through river land-based sources, it is an indispensable nutrient source for diatoms [54,55]. Moreover, the variation range of environmental factors in spring and autumn is more intense than in summer and winter. The zooplankton diversity increases rapidly with a rise in the water temperature and improvement in nutrition. Alternatively, in the autumn, the environmental factors became so barren that the biodiversity index quickly slid off. Moreover, WT, Sal, pH and Chl-a in summer are the main factors possibly affecting the changes in the biological community in summer. On the contrary, the dissolved inorganic nutrients in winter were significantly different from those in other seasons. The results of the RDA revealed the relationship between the environmental factors and the seasonal structure of zooplankton. Water temperature was still the most important factor influencing the spatial and temporal distribution pattern of zooplankton assemblages in subtropical marine areas. In spring and summer, DO and Sal showed a positive correlation with water temperature for the zooplankton (Figure 5b,c). Winter and autumn were just the opposite of the coordinate axis of WT in spring and summer, and DO become the most important correlated factor (Figure 5d,e), suggesting that a change in DO has a great impact on the biological community under the state of low temperature. Abdul et al. (2016) and Basu et al. (2022) both recognized that water variables (temperature, salinity, transparency and DO) significantly explain the principal variations in the zooplankton species composition in the coastal estuary, which was in line with the present results [39,56]. Finally, the spatial variation in the water temperature was considerable (Table 1). We speculated that the discharge of power plants reported in the surrounding area had a direct effect [57]. Luckily, the spatial difference in the community structure was slight, indicating that the connectivity of zooplankton among different sites was good. The impact of wastewater discharge and other industrial development on these marine organisms was not detected yet. Indeed, the impact of human activities on aquatic life is increasingly concerning [58,59,60]. Zooplankton, as an important environmental health indicator, can provide information on environmental changes and impacts by monitoring their community structures [61]. In the future, it is still necessary to further strengthen monitoring and management, improve the frequency of investigation and increase the laboratory simulation study of the zooplankton community to provide biodiversity protection for the surrounding sea.

## 5. Conclusions

The present study presented the composition and distribution of zooplankton assemblages from the surrounding area of Tiaowei Island, an island near a small county under rapid development. Dominated by Copepoda, the assemblage of 116 taxa in total is considered a relatively high richness, abundance and evenness. More than 20 species of Medusae were observed, suggesting a potential risk of biological disasters. Significant seasonal variations rather than spatial differences were detected in both the zooplankton structure and environmental variables. The RDA revealed that water temperature, pH, salinity, DO and suspended particulate composition were the main environmental variants to seasonally impact the zooplankton communities. In terms of the organisms, planktonic larvae and some Copepoda species showed abundances correlated with the changes in environmental variants. This indicates that these taxa can be used as indicators for monitoring the local marine environment. Finally, despite the rapid industrial development around the coastal area in recent years, no rapid feedback from the zooplankton communities has been found. It is still necessary to further strengthen monitoring and management, improve the frequency of investigation, and increase the laboratory simulation study on the zooplankton community to provide biodiversity protection for the surrounding sea.

## Figures and Tables

**Table 1:** Variations in physical and chemical characteristics in ten cruises.

	Jan-2018	Apr-2018	Jul-2018	Oct-2018	Jan-2019	Apr-2019	Jul-2019	Apr-2020	Oct-2020	Dec-2020
WT (℃)	13.08 ± 1.21 (12.2~15.8)	20.12 ± 2.11 (17.9~24.8)	31.40 ± 2.16 (29.8~37.6)	20.88 ± 0.67 (20.1~21.8)	12.76 ± 0.41 (12.4~13.6)	17.32 ± 0.88 (16.4~19.5)	30.36 ± 0.43 (29.8~31.5)	18.33 ± 1.47 (17.3~22.6)	24.68 ± 1.90 (23.6~28.8)	19.87 ± 3.21 (16.0~24.7)
Sal (PSU)	26.90 ± 0.15(26.65~27.25)	29.46 ± 0.20(29.11~29.72)	32.48 ± 0.22 (32.22~32.86)	27.96 ± 0.32 (27.41~28.40)	27.17 ± 0.20 (27.03~27.78)	27.55 ± 0.16 (27.22~27.72)	30.60 ± 0.16 (30.35~30.86)	27.70 ± 0.14 (27.24~27.78)	28.02 ± 0.37 (27.50~28.41)	25.94 ± 0.04 (25.87~26.02)
pH	8.04 ± 0.02 (8.00~8.06)	8.13 ± 0.03 (8.08~8.17)	8.15 ± 0.04 (8.10~8.22)	7.99 ± 0.04 (7.94~8.06)	8.10 ± 0.01 (8.08~8.11)	8.07 ± 0.04 (8.03~8.14)	8.13 ± 0.02 (8.10~8.16)	8.11 ± 0.02 (8.07~8.13)	8.12 ± 0.01 (8.10~8.13)	8.11 ± 0.07 (7.97~8.20)
DO (μmol·L^−1^)	9.09 ± 0.15 (8.81~9.30)	8.09 ± 0.36 (7.50~8.60)	7.52 ± 0.77 (6.36~8.65)	7.17 ± 0.25 (6.73~7.46)	8.82 ± 0.10 (8.64~8.94)	7.88 ± 0.38 (7.40~8.58)	5.81 ± 0.25 (5.53~6.37)	7.86 ± 0.12 (7.73~8.14)	7.23 ± 0.34 (6.38~7.84)	6.84 ± 0.16 (6.54~7.17)
COD (mg·L^−1^)	1.18 ± 0.26 (0.89~1.94)	0.78 ± 0.11 (0.62~0.97)	0.95 ± 0.24 (0.63~1.39)	0.75 ± 0.25 (0.50~1.42)	1.13 ± 0.26 (0.75~1.56)	0.84 ± 0.32 (0.54~1.75)	0.85 ± 0.20 (0.48~1.16)	0.71 ± 0.10 (0.59~0.86)	1.10 ± 0.32 (0.5~1.6)	0.94 ± 0.47 (0.47~1.76)
PO_4_—P (nmol·L^−1^)	0.026 ± 0.009(0.014~0.043)	0.014 ± 0.004 (0.008~0.021)	0.017 ± 0.004 (0.009~0.022)	0.031 ± 0.011 (0.015~0.047)	0.055 ± 0.010 (0.047~0.074)	0.021 ± 0.008 (0.012~0.033)	0.015 ± 0.002 (0.013~0.019)	0.033 ± 0.001 (0.030~0.035)	0.021 ± 0.003 (0.017~0.027)	0.032 ± 0.006 (0.026~0.040)
NO_2_—N (nmol·L^−1^)	0.007 ± 0.003 (0.003~0.017)	0.007 ± 0.001 (0.005~0.009)	0.010 ± 0.004 (0.001~0.017)	0.008 ± 0.00 (0.005~0.01)	0.005 ± 0.001 (0.004~0.006)	0.008 ± 0.004 (0.003~0.014)	0.017 ± 0.002 (0.015~0.020)	0.016 ± 0.001 (0.014~0.018)	0.010 ± 0.005 (0.006~0.018)	0.008 ± 0.002 (0.006~0.013)
NO_3_—N (nmol·L^−1^)	0.35 ± 0.11 (0.19~0.56)	0.22 ± 0.06 (0.12~0.30)	0.07 ± 0.02 (0.03~0.10)	0.36 ± 0.14 (0.18~0.57)	0.54 ± 0.02 (0.51~0.56)	0.33 ± 0.1178 (0.18~0.48)	0.10 ± 0.00 (0.09~0.12)	0.52 ± 0.02 (0.48~0.55)	0.23 ± 0.05 (0.15~0.31)	0.61 ± 0.02 (0.59~0.64)
NH_4_—N (nmol·L^−1^)	0.026 ± 0.014 (0.013~0.065)	0.036 ± 0.035 (0.013~0.114)	0.021 ± 0.006 (0.015~0.039)	0.014 ± 0.004 (0.007~0.020)	0.039 ± 0.016 (0.022~0.079)	0.031 ± 0.013 (0.018~0.063)	0.015 ± 0.005 (0.008~0.024)	0.031 ± 0.009 (0.018~0.048)	0.031 ± 0.017 (0.017~0.080)	0.048 ± 0.016 (0.018~0.069)
DIN (nmol·L^−1^)	0.39 ± 0.11 (0.21~0.60)	0.27 ± 0.09 (0.16~0.27)	0.10 ± 0.03 (0.05~0.15)	0.38 ± 0.14 (0.19~0.60)	0.59 ± 0.02 (0.56~0.65)	0.37 ± 0.12 (0.21~0.52)	0.13 ± 0.01 (0.11~0.16)	0.57 ± 0.02 (0.52~0.59)	0.27 ± 0.06 (0.19~0.39)	0.67 ± 0.01 (0.63~0.69)
SiO_4_-Si (nmol·L^−1^)	0.77 ± 0.25 (0.38~1.27)	0.46 ± 0.11 (0.27~0.62)	0.47 ± 0.09 (0.26~0.58)	0.77 ± 0.34 (0.25~1.22)	1.30 ± 0.03 (1.22~1.33)	0.66 ± 0.025 (0.34~0.98)	0.66 ± 0.06 (0.54~0.76)	NA	NA	NA
Chl-a (μg·L^−1^)	0.45 ± 0.15 (0.24~0.68)	5.82 ± 2.20 (1.36~9.00)	3.03 ± 1.12 (1.17~5.67)	0.81 ± 0.29 (0.44~1.56)	0.61 ± 0.01 (0.46~0.77)	1.37 ± 0.41 (0.56~2.24)	5.69 ± 2.53 (1.8~10.33)	0.93 ± 0.18 (0.68~1.15)	5.02 ± 1.42 (2.73~6.62)	1.03 ± 0.36 (0.65~1.56)
ss (mg·L^−1^)	93.69 ± 20.39 (48.2~133.0)	26.92 ± 12.89 (11.6~53.4)	19.43 ± 6.03 (12.4~33.2)	44.60 ± 12.65 (27.0~69.2)	144.39 ± 38.42 (96.0~212.3)	57.20 ± 27.779 (20.8~95.2)	43.85 ± 12.81 (18.8~63.0)	29.40 ± 9.21 (18.4~52.8)	119.93 ± 100.3 (25.6~383.2)	152.43 ± 173.1 (25.6~528.7)

Table 1 shows the means and standard deviations of environmental parameters for each cruise. The numbers in brackets are the range in which the feature varies. NA means not analyzed; WT, Sal and ss stand for water temperature, salinity and suspended particulate composition, respectively.

**Table 2:** Variations in species number and diversity indices from (**a**) different cruises and (**b**) stations.

**(a)**	Jan-2018	Apr-2018	Jul-2018		Oct-2018	Jan-2019	Apr-2019	Jul-2019	Apr-2020		Oct-2020	Dec-2020
*S*	22	43	80		45	33	40	34	46		46	55
*D*	4.702	7.883	12.411		8.059	6.290	7.084	6.375	8.329		8.408	9.987
*J*′	0.9039	0.9303	0.9156		0.9354	0.9165	0.9399	0.9173	0.9331		0.9350	0.9142
*H’*	2.794	3.499	4.012		3.561	3.205	3.467	3.235	3.572		3.580	3.664
**(b)**	X5	X6	X7	X10	X12	X14	X17	X19	X21	X22	X23	X24
*S*	78	65	69	73	69	71	66	63	64	61	68	54
*D*	14.44	12.20	12.92	13.64	13.27	13.52	12.56	12.09	12.16	11.67	12.92	11.28
*J’*	0.9489	0.9381	0.9456	0.9476	0.9481	0.9367	0.9370	0.9472	0.9426	0.9354	0.9324	0.9593
*H’*	4.134	3.916	4.004	4.066	4.014	3.993	3.926	3.925	3.920	3.846	3.934	3.827

*S*, *D*, *J*′ and *H*′ represent taxa richness, Margalef richness index, Pielou’s evenness index, and Shannon–Wiener diversity index, respectively.

## Data Availability

Data may be provided on request to the corresponding author.
